# Plasma Homocysteine, Vitamin B12 and Folate Levels in Multiple System Atrophy: A Case-Control Study

**DOI:** 10.1371/journal.pone.0136468

**Published:** 2015-08-20

**Authors:** Shuyu Zhang, Changhe Shi, Chengyuan Mao, Bo Song, Haiman Hou, Jun Wu, Xinjing Liu, Haiyang Luo, Shilei Sun, Yuming Xu

**Affiliations:** Department of Neurology, The first affiliated Hospital of Zhengzhou University, Zhengzhou University, Zhengzhou, Henan, China; Fatebenefratelli Foundation for Health Research and Education, ITALY

## Abstract

**Background:**

Multiple system atrophy (MSA) is a neurodegenerative disease, and its pathological hallmark is the accumulation of α-synuclein proteins. Homocysteine (Hcy) is an intermediate amino acid generated during the metabolism of methionine. Hcy may contribute to the pathogenesis of neurodegenerative disorders. Vitamin B12 and folate are cofactors necessary for the methylation of homocysteine.

**Methods:**

This study compared the levels of serum Hcy, vitamin B12 and folate in patients with MSA with those in healthy people to reveal the possible association between MSA and plasma levels of Hcy, vitamin B12 and folate. We enrolled 161 patients with MSA and 161 healthy people in this study. The association between MSA and the levels of Hcy, vitamin B12 and folate were analyzed using binary logistic regression.

**Results:**

The mean level of Hcy in patients with MSA was significantly higher than that in healthy controls (16.23 ± 8.09 umol/l vs 14.04 ± 4.25 umol/l, p < 0.05). After adjusting for age, sex and medical history, the odds ratio for Hcy was 1.07 (95% CI = 1.01–1.13, p < 0.05) for patients with MSA. Vitamin B12 and folate levels were not significantly different between patients with MSA and controls.

**Conclusion:**

Our data suggest that higher levels of Hcy may be associated with an increased risk for MSA.

## Introduction

Multiple system atrophy (MSA) is a sporadic neurodegenerative disease characterized by the symptoms of Parkinson's syndrome, autonomic nervous system dysfunction, cerebellar ataxia, and pyramidal tract symptom [1]. MSA is divided into three types according to the onset sign and the severity of different symptoms: the parkinsonian type (MSA-P), the cerebellar type (MSA-C), and the autonomic type (MSA-A). The annual incidence of MSA has been estimated about 4.4 cases per 100,000 persons at the age of 50 to 99 [2]. The prognosis of MSA patients was poor with survival time of less than 9 years on average [3,4]. Single nucleotide polymorphisms (SNPs) at the SNCA locus were associated with increased risk of MSA [5]. Even though previous studies have concluded that the ectopic appearance of α-synuclein in oligodendrocytes, oxidative stress, mitochondrial dysfunction, and energy failure may contribute to the pathogenesis of MSA [6,7], the mechanism of MSA is still unknown. Moreover currently there is no effective treatment for MSA, and the disease imposes an immense economic burden and psychological pressure on the individuals affected and on the society. So the early diagnosis and treatment are very important, and it’s necessary to looking for biomarkers of MSA.

Homocysteine (Hcy) is an intermediate of methionine metabolism, and its level is kept low by its conversion to cysteine [8]. Vitamin B12 and folate play an important role in this conversion. Previous studies have described that Hcy may contribute to the pathogenesis of neurodegenerative disorders via the increase of oxidative injury in neurons, stimulation of *N*-methyl-D-aspartate (NMDA) receptors [9–11]. And Hcy can induce neuronal apoptosis and can increase neuronal vulnerability to excitotoxicity by a mechanism involving DNA strand breakage, poly-ADP-ribose polymerase (PARP) activation, and p53 induction [9]. Moreover, a randomized controlled study revealed a positive correlation between the levels of Hcy and the rate of brain atrophy [12]. Some studies have investigated the relationship between plasma Hcy levels in patients with Parkinson’s disease (PD) or Alzheimer’s disease, and demonstrated that Hcy play a potential role in the neurodegenerative disorders [13]. Nevertheless, there were no any studies about the Hcy levels in MSA patients. This study aimed to evaluate the levels of plasma Hcy, vitamin B12, and folate in MSA to substantiate future etiological studies.

## Methods

We enrolled 161 patients at the First Affiliated Hospital of Zhengzhou University of Henan province from January 2011 to September 2014. Patients were diagnosed and divided into three subtypes (MSA-P, MSA-C, MSA-A) by two neurologists based on the consensus criteria for the clinical diagnosis of MSA which established in 2008 [7]. Patients with a sporadic, progressive adult-onset disorder including strictly defined autonomic dysfunction and poorly levodoparesponsive parkinsonism or cerebellar ataxia were diagnosed with probable MSA, and patients with a sporadic, progressive adult-onset disease including parkinsonism or cerebellar ataxia and at least one feature of autonomic dysfunction plus one other feature that may be a clinical or a neuroimaging abnormality were diagnosed with possible MSA. The clinical severity of MSA was defined according to the Hoehn and Yahr (H&Y) stage [14]. For the control group, we selected 161 age- and sex-matched healthy people who visited the hospital for physical examination in the same period. People in the control group were free of any neurological diseases, cardiopathy, hypothyroidism, severe liver or kidney disease. Enrolled patients and controls were not treated with vitamin B12 or folate. Basic information including sex, age, history of smoking, drinking, hypertension, diabetes mellitus, and data of Hcy, vitamin B12, and folate levels were collected retrospectively from the medical records. The presence or absence of dysphagia was determined in patients. The study had been approved by the First Affiliated Hospital of Zhengzhou University Ethics Committee. Written informed consent was obtained from all participants.

Continuous variables were presented as mean ± standard deviation. Categorical variables were presented as percentages. t and χ^2^ tests were used to compare the demographic features of patients with MSA and controls, and Bonferroni correction was used in subgroup analysis. After adjusting for the history of smoking, drinking, hypertension, and diabetes mellitus, odds ratio (OR) and the 95% confidence interval (CI) were estimated by logistic regression. Statistical analyses were performed using the SPSS 16.0 software, all tests were two-tailed and p < 0.05 was considered as statistically significant.

## Results

Baseline data are presented in Table 1. Among the 161 patients with MSA, 82 were male and 79 were female. There was no significant difference in the age of patients (57.99 ± 8.34, years) and controls (57.34 ± 10.37, years). Similarly, we did not find any significant difference in sex distribution, history of smoking, and diabetes mellitus. History of drinking and hypertension were more frequently observed in patients with MSA than in controls (drinking: 12.42% vs 3.11%, p < 0.05. hypertension: 22.36% vs 6.83%, p < 0.05). A total of 35 patients suffered from dysphagia (21.12%). H&Y stage were available in each patient. H&Y stage, mean disease duration, and onset age in patients were 3.06 ± 1.07, 2.35 ± 2.07 years and 55.63 ± 8.50 years old, respectively. The Hcy levels were higher than the upper limit of the reference range (5–15 umol/l) in 49 patients. Moreover, the mean Hcy level in patients with MSA was significantly higher than that in controls (16.23 ± 8.09 vs 14.04 ± 4.25 umol/l, p<0.05). There were no significant differences in the levels of folate and vitamin B12 between the two groups. In sex-specific comparisons, hypertension was observed significantly more often in patients with MSA than it was in controls in both male (20.73% vs 7.69%, p < 0.05) and female (24.05% vs 6.02%, p < 0.05) individuals; however, drinking history was observed more frequently in male with MSA than it was in controls (24.39% vs 3.11%, p < 0.05). The mean level of Hcy in patients with MSA was significantly higher than that in controls in male (18.06 ± 8.16 umol/l vs 15.02 ± 4.32 umol/l, p < 0.05). However, the level of Hcy was not significantly different between patients and controls in all women (p > 0.05). Folate and vitamin B12 levels were not statistically different in the patient and control groups of male or female individuals. In subgroup analysis, Hcy, folate and vitamin B12 levels were not significantly different among the three subtype patients with MSA and controls (Table 2).

**Table 1:** Characteristics of patients and controls.

	Patients(161)			Controls(161)			P		
	all	Men(82)	Women(79)	all	Men(78)	Women(83)	P _all_	P _m_	P _w_
Age (years)	57.99±8.34	57.34±8.43	58.67±8.25	57.34±10.37	58.68±9.17	56.08±11.30	NS	NS	NS
Dysphagia (%)	34(21.12)	16(19.51)	18(22.78)	—	—	—	—	—	—
Smoking (%)	20(12.42)	20(24.39)	0(0)	15(9.32)	15(9.32)	0(0)	NS	NS	—
Drinking(%)	20(12.42)	20(24.39)	0(0)	5(3.11)	5(3.11)	0(0)	<0.05	<0.05	—
Hypertension (%)	36(22.36)	17(20.73)	19(24.05)	11(6.83)	6(7.69)	5(6.02)	<0.05	<0.05	<0.05
Diabetes mellitus (%)	14(8.70)	8(9.76)	6(7.59)	14(8.70)	5(6.41)	9(10.84)	NS	NS	NS
Hcy (umol/l)	16.23±8.09	18.06±8.16	14.24±7.60	14.04±4.25	15.02±4.32	13.12±4.01	<0.05	<0.05	NS
Folate (ng/ml)	6.46±3.14	6.11±3.06	6.80±3.23	6.40±3.28	6.06±3.31	6.73±3.25	NS	NS	NS
Vitamin B12 (pg/ml)	600.85±515.69	559.50±468.63	644.13±563.07	547.1±479.56	541.80±459.40	552.40±501.82	NS	NS	NS
Onset age (years)	55.63±8.50	54.82±8.55	56.47±8.42	—	—	—	—	—	—
Disease duration (years)	2.35±2.07	2.47±2.16	2.23±1.99	—	—	—	—	—	—
H&Y	3.06±1.07	2.95±1.08	3.16±1.06	—	—	—	—	—	—

H & Y: Hoehn and Yare stage; P _all_, comparison between MSA patients and controls in all participants; P_m_, comparison between MSA patients and controls in male; P_w_, comparison between MSA patients and controls in female; NS, not significant.

**Table 2:** Comparison among the different subtypes patients and controls.

	Patients(161)			Controls(161)	P					
	MSA-C(91)	MSA-P(53)	MSA-A(17)		P_c_	P_p_	P_a_	P_cp_	P_ca_	P_pa_
Age (year)	56.59±8.73	58.89±7.14	62.71±8.01	57.34±10.37	NS	NS	NS	NS	NS	NS
Hcy (umol/l)	16.70±9.55	16.06±4.75	13.95±7.03	14.04±4.25	NS	NS	NS	NS	NS	NS
Folate (ng/ml)	6.12±3.12	6.82±3.30	7.83±2.57	6.40±3.28	NS	NS	NS	NS	NS	NS
Vitamin B12(pg/ml)	617.17±551.40	538.65±407.41	681.24±597.09	547.1±479.56	NS	NS	NS	NS	NS	NS

P_c_, P_p_, P_a_: comparison between controls and MSA-C, MSA-P, MSA-A patients, respectively, P_cp_: comparison between MSA-C and MSA-P, P_ca_: comparison between MSA-C and MSA-A, P_pa_: comparison between MSA-P and MSA-A, NS, not significant.

Univariate analysis showed that the OR of Hcy was 1.06 (95% CI = 1.02–1.11, p < 0.05) for patients with MSA, but the OR of folate and vitamin B12 were not statistically significant. In sex-specific comparisons, the OR of Hcy was 1.08 (95% CI = 1.02–1.15, p < 0.05) in the male group, the OR of Hcy in the female group and the OR of folate and vitamin B12 in the female and male groups were not significant. After adjusting for age, sex, history of smoking, drinking, hypertension and diabetes mellitus, the OR of Hcy was 1.07 (95% CI = 1.01–1.13, p < 0.05) for patients with MSA.

**Fig 1:**
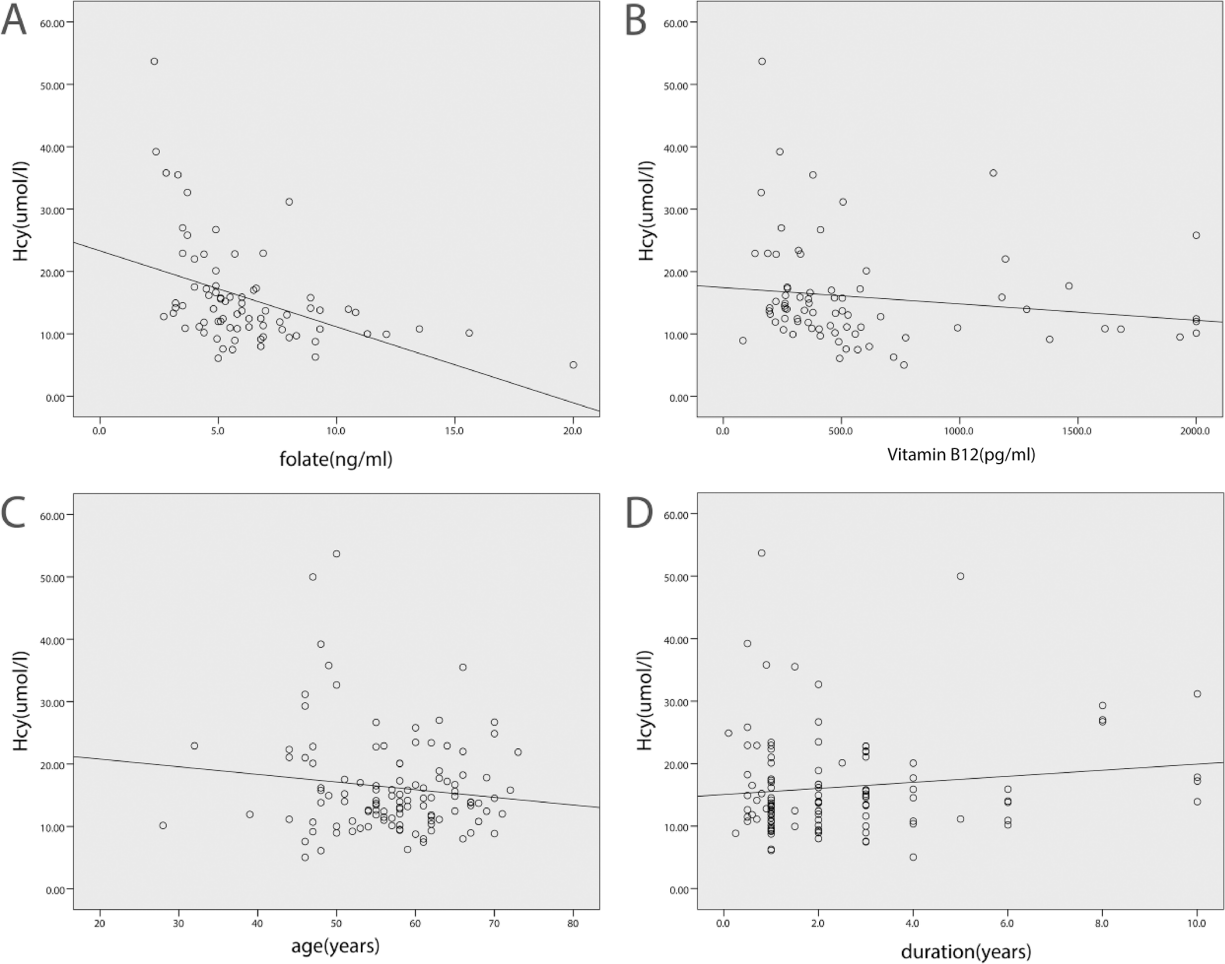
Correlation analysis between Hcy and folate, vitamin B12, age, disease duration respectively in MSA patients. (A): Hcy levels were significantly negative correlate with folate in MSA patients; (B): Hcy levels were significantly negative correlate with vitamin B12 in MSA patients; (C): Hcy levels were not significantly correlate with age in MSA patients; (D): Hcy levels were not significantly correlate with disease duration in MSA patients.

The levels of Hcy were in a significantly negative correlation with folate (r = -0.42, p < 0.05) and vitamin B12 (r = -0.45, p < 0.05) in all individuals, and the levels of Hcy did not correlate with age (r = -0.06, p > 0.05). Correlation analysis in patients also showed that the levels of Hcy were in a significantly negative correlation with folate (r = -0.50, p < 0.05) and vitamin B12 (r = -0.33, p < 0.05). Hcy levels did not correlate with age (r = -0.03, p > 0.05), disease duration (r = 0.06, p > 0.05), onset age (r = -0.06, p > 0.05), or H&Y stage (r = -0.01, p > 0.05) in patients with MSA. (Fig 1.)

## Discussion

To the best of our knowledge, this is the first study investigating the correlation between the levels of plasma Hcy, vitamin B12, and folate in patients with MSA. We demonstrated that patients suffering from MSA have higher levels of Hcy compared to healthy people, however, vitamin B12 and folate levels were not statistically different between the two groups. After adjusting for age, sex, history of smoking, drinking, hypertension, and diabetes mellitus, we demonstrated patients with MSA have higher level of Hcy than healthy people. The mean level of Hcy in male patients with MSA was significantly higher compared to that in male controls. We found no statistically significant difference in the mean level of Hcy between the controls and patients in female, but there is a tendency that the level of Hcy in female patients was higher than that in the female controls. The analysis of MSA subtypes revealed that Hcy Vitamin B12 and folate levels were not statistically different.

MSA and PD are progressive neurodegenerative disorders characterized neuropathologically by the abnormal deposition of α-synuclein [15]. In the earliest stages of MSA, pathogenesis may involve the over-expression or aberrant accumulation of α-synuclein in oligodendrocytes [16,17]. Oxidative stress is a well-known factor that contributes to the aggregation of α-synuclein [18]. Hcy may cause calcium overload and glutamate excitotoxicity by stimulating NMDA receptors in the cortical neurons [9,13]. In addition, Hcy may overstimulate the poly ADP-ribosome polymerase (PARP) and therefore contribute to neuronal oxidative stress and apoptosis by damaging DNA [9]. These studies showed that Hcy hastened the progression of PD by increasing the susceptibility of dopaminergic neurons [19], and Hcy is promoting oxidative stress by auto-oxidation [20]. Therefore, we speculated that Hcy level may be associated with MSA. Hcy is a risk factor for cardiovascular disease, because it leads to insufficient blood supply by causing an injury to the vascular endothelium, and in addition, it weakens the synthesis of synapses and causes nerve degeneration. Vitamin B12 and folate are crucial cofactors for the methylation of Hcy. In this study, Hcy levels in all people or in patients with MSA only were in a significantly negative correlation with vitamin B12 and folate levels [9,21,22]. The levels of vitamin B12 and folate were not statistically different between patients and controls in this study. A possible explanation for this finding is that unknown factors might regulate the level of folate and vitamin B12 in patients with MSA. Taking our findings and the literature into consideration, we speculate that Hcy may play a possible role in the pathogenesis of MSA.

This study revealed some interesting results that require discussion. The ratio of patients with a history of excessive alcohol consumption was higher than that in controls, and mainly male patients were affected. Previous studies did not establish a relationship between drinking and MSA, and there is only one meta-analysis showing that alcohol intake, especially beer, might be inversely associated with the risk of PD [23]. To confirm the association between alcohol consumption and MSA, well designed prospective studies will be required. In this study, we found that Hcy level was higher in male than it was in female, and not only in patients with MSA, but in controls, too. This verified the views that the lack of estrogen can cause an increase in the level of Hcy, and the folate metabolism is different between individuals of different sex [24]. Environmental factors such as excessive coffee or alcohol intake, smoking, or physical inactivity may increase the plasma levels of Hcy [25]. In our study, a history of drinking was observed more frequently in men. We showed that the increased Hcy level is more common in male than it is in female, so we assume that men are more likely to suffer from hyperhomocysteinemia, and therefore the risk of MSA is higher in male. However, additional clinical investigations are necessary to confirm our hypothesis.

Some limitations of the study need to be addressed. First, this was a case-control study, and therefore data should be interpreted with caution because we did not investigate the longitudinal effects of Hcy level on the development of MSA. Second, we found that the level of Hcy did not correlate with age. This finding contradicts the view that the level of Hcy is increased with age, because the levels of folate and vitamins are reduced in the elderly [26].

In summary, our results showed that Hcy may play a possible role in the pathogenesis of MSA and may increase the risk of MSA. Considering the burden of MSA, it is warranted to perform prospective investigations to confirm our hypothesis.

## Supporting Information

S1 Data Supporting information file (primary data). (XLS)
